# Efficacy of breast MRI for surgical decision in patients with breast cancer: ductal carcinoma in situ versus invasive ductal carcinoma

**DOI:** 10.1186/s12885-020-07443-7

**Published:** 2020-09-29

**Authors:** Jeeyeon Lee, Jin Hyang Jung, Wan Wook Kim, Chan Sub Park, Ryu Kyung Lee, Hye Jung Kim, Won Hwa Kim, Ho Yong Park

**Affiliations:** 1 Department of Surgery, School of Medicine, Kyungpook National University, Daegu, Republic of Korea; 2 Department of Radiology, School of Medicine, Kyungpook National University, Daegu, Republic of Korea; 3 Department of Surgery, Joint Institute for Regenerative Medicine, School of Medicine, Kyungpook National University, Hoguk-ro 807, Buk-gu, Daegu, 41404 Republic of Korea

**Keywords:** Breast, Ductal carcinoma, Magnetic resonance imaging, Surgical plan

## Abstract

**Background:**

Preoperative breast magnetic resonance imaging (MRI) provides more information than mammography and ultrasonography for determining the surgical plan for patients with breast cancer. This study aimed to determine whether breast MRI is more useful for patients with ductal carcinoma in situ (DCIS) lesions than for those with invasive ductal carcinoma (IDC).

**Methods:**

A total of 1113 patients with breast cancer underwent mammography, ultrasonography, and additional breast MRI before surgery. The patients were divided into 2 groups: DCIS (*n* = 199) and IDC (*n* = 914), and their clinicopathological characteristics and oncological outcomes were compared. Breast surgery was classified as follows: conventional breast-conserving surgery (Group 1), partial mastectomy with volume displacement (Group 2), partial mastectomy with volume replacement (Group 3), and total mastectomy with or without reconstruction (Group 4). The initial surgical plan (based on routine mammography and ultrasonography) and final surgical plan (after additional breast MRI) were compared between the 2 groups. The change in surgical plan was defined as group shifting between the initial and final surgical plans.

**Results:**

Changes (both increasing and decreasing) in surgical plans were more common in the DCIS group than in the IDC group (*P* <  0.001). These changes may be attributed to the increased extent of suspicious lesions on breast MRI, detection of additional daughter nodules, multifocality or multicentricity, and suspicious findings on mammography or ultrasonography but benign findings on breast MRI. Furthermore, the positive margin incidence in frozen biopsy was not different (*P* = 0.138).

**Conclusions:**

Preoperative breast MRI may provide more information for determining the surgical plan for patients with DCIS than for those with IDC.

## Background

Preoperative breast magnetic resonance imaging (MRI) is an optional modality for the evaluation of breast cancer. However, compared with mammography or ultrasonography, it can provide additional information for diagnosing ductal carcinoma in situ (DCIS) [1–3]. In addition, the involvement of the nipple or nipple-areolar complex in breast cancer can be easily detected with additional breast MRI [4, 5]. The usefulness of breast MRI has been demonstrated among Asian women who have dense breasts or are BRCA mutation carriers with a higher risk of contralateral breast cancer [6, 7].

The surgical plan for breast cancer is usually determined according to the excision volume, tumor location, glandular density, and ratio of tumor to whole breast volume [8, 9]. The tumor extent, ductal pattern, existence of daughter nodules, and multifocality or multicentricity can be detected in additional breast MRI. However, because the characteristics of images differ depending on the tumor type, the imaging modality to be performed should be carefully determined. Although DCIS has excellent prognosis, the excision volume is usually larger than that of a single nodule of invasive ductal carcinoma (IDC) due to the ductal pattern [2, 10]. In triple-negative breast cancer, the breast lesion may appear round, which can be misinterpreted as a benign lesion [11–13]. In those cases, additional preoperative breast MRI can provide more important information.

In this study, we evaluated the usefulness of preoperative breast MRI in determining the surgical plan for patients with breast cancer [14]. Although this result was not described in detail in our previous study, we found that changes in surgical plans were more common among patients with carcinoma in situ lesions than among those with invasive carcinoma (13.0% vs. 9.9%). Hence, this study aimed to determine whether breast MRI is more useful in determining the surgical plan for patients with DCIS than for those with IDC.

## Methods

**Fig. 1:**
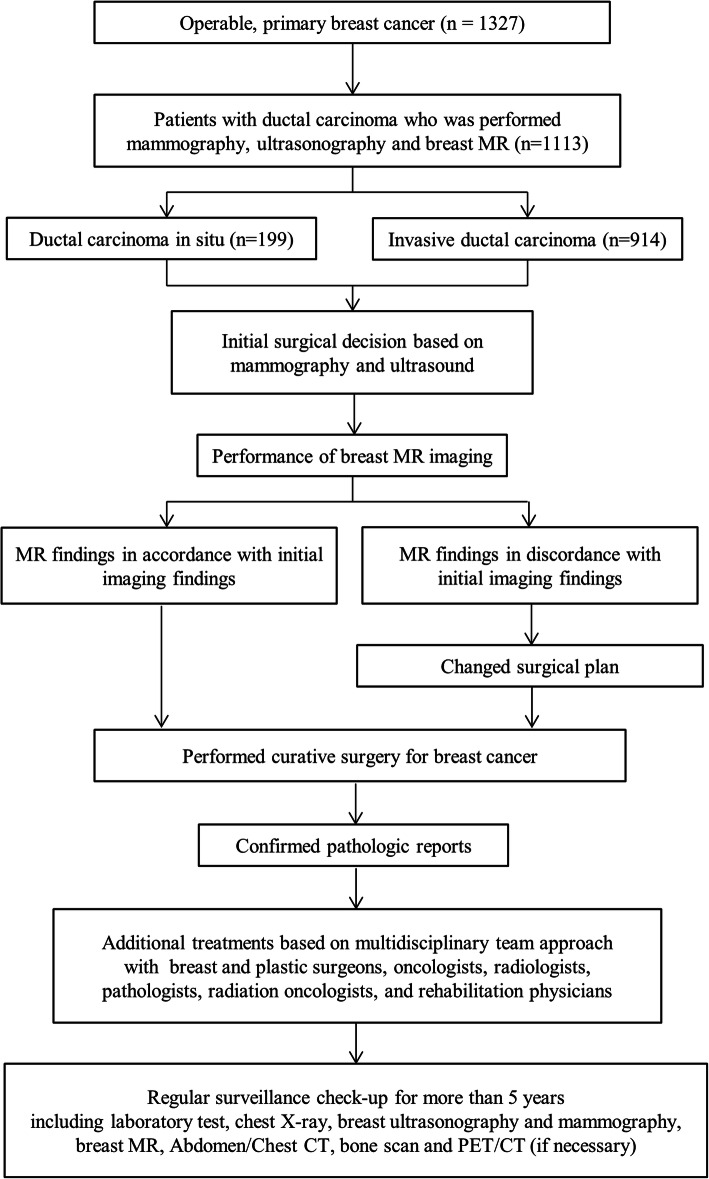
A flowchart demonstrating the process of changing surgical plans based on the results of mammography, ultrasonography, and additional breast magnetic resonance imaging, as well as the management of breast cancer using a multidisciplinary team approach

Between 2006 and 2014, the medical records and oncological status of 1327 patients with operable, primary breast cancer who underwent cancer surgery at Kyungpook National University Hospital (Daegu, Republic of Korea) were reviewed. Among them, 1113 patients with ductal carcinoma underwent mammography, breast ultrasonography, and MRI before surgery. Breast MRI was performed with the patient prone using a 1.5 T system (Signa Excite; GE Medical Systems, Milwaukee, WI) with a dedicated 4-channel breast coil. Each patient was given 0.1 mL/kg gadolinium-diethylenetriamine pentaacetate (Magnevist; Schering, Berlin, Germany) as the contrast agent, which was injected at a rate of 1 mL/s. Axial T1-weighted images (repetition time [TR]/echo time [TE], 416/10; matrix, 320 × 224; slice thickness, 3.4 mm) and sagittal fat-suppressed T2-weighted images (TR/TE, 3000/94; matrix, 320 × 224; slice thickness, 2.6 mm) were acquired. Dynamic contrast-enhanced magnetic resonance examination included 1 precontrast and 5 postcontrast images with bilateral sagittal acquisition by 3-dimensional gradient-echo fat-suppressed T1-weighted imaging (TR/TE, 6.2/2.9; matrix, 288 × 416; flip angle, 10°; slice thickness, 2.6 mm). ​The patients were divided into 2 groups (DCIS and IDC) based on needle biopsy results (Fig. 1). The study protocol was approved by the Institutional Review Board Committee of Kyungpook National University Hospital (2016–10-008).

The treatment for breast cancer was determined by the combined opinion of a multidisciplinary team including breast and plastic surgeons, oncologists, radiologists, pathologists, and radiation oncologists. Based on the resection volume of the breast, the types of surgery were classified as follows: breast-conserving surgery (Group 1), partial mastectomy with volume displacement (Group 2), partial mastectomy with volume replacement (Group 3), and total mastectomy with or without breast reconstruction (Group 4).

The initial surgical plan was determined based on mammography and ultrasonography findings, and the final surgical plan was determined based on additional breast MRI findings and second-look ultrasonography results when an additional suspicious lesion was detected on the breast MRI. When an additional suspicious lesion (detected on breast MRI and second-look ultrasonography) was present in different quadrants of the breast, biopsy was performed. Both surgical plans were compared to determine whether the interventions involved were the same. The change in surgical plan was defined as group shifting between the initial and final surgical plans.

The inclusion criteria for this study were as follows: 1) Biopsy revealed malignant disease with ductal origin; and 2) additional breast MRI was conducted before surgery and deciphered by highly experienced radiologists. Patients whose biopsy result was reported as other malignant breast diseases excluding ductal carcinoma, who developed other primary malignancies, and who underwent neoadjuvant chemotherapy or excisional biopsy before breast MRI were excluded. Regardless of DCIS or IDC, when the final diagnosis after surgery was different from the initial diagnosis, those patients were also excluded. In addition, if an extensive intraductal component was found based on the result of needle biopsy for IDC, those patients were excluded to reduce bias. For the evaluation of the surgical margin status, breast tissues were obtained in at least four different directions from the cavity and used to prepare frozen sections and permanent sections. Surgical margin positivity was defined as the presence of atypical cells, carcinoma in situ, or invasive cancer cells on the cut surface. After the negative results of the surgical margin were confirmed, reconstructive surgery was performed.

Patients who requested to change their surgical plan regardless of imaging findings or those whose surgical plan was changed because of the previous excision status (not because of breast MRI findings) were included in the patient group with changes in surgical plans but were excluded from the group when changes in surgical plans were related to MRI results. Additional treatments including adjuvant chemotherapy, radiotherapy, hormone therapy, or targeted treatment were administrated based on the patients’ tumor characteristics.

The follow-up period was extended to at least 4 years after the initial treatment. The oncological outcomes were evaluated to determine the rate of locoregional recurrence, distant metastasis, and mortality. Regular surveillance was performed every 6 months during the first 2 years and annually after 3 years, and included laboratory blood test with tumor markers, chest x-ray, mammography, breast ultrasonography, thoracic and abdominal computed tomography, bone scan, and positron emission tomography/computed tomography (if necessary).

The clinicopathological factors were obtained from medical records, and statistical analysis was performed using SPSS (version 23; SPSS Inc., Chicago, IL, USA). Quantitative and categorical variables were compared using Student’s *t* test and the χ^2^ test, respectively. A *P* value of less than 0.05 was considered significant.

## Results

There were no significant differences in the mean age, mean body mass index, and clinical and pathological tumor size between the DCIS and IDC groups. The incidence of bilateral breast cancer was higher in the DCIS group (DCIS, 7.0% vs. IDC, 3.3%; *P* = 0.014), and triple-negative breast cancer was more frequent in the IDC group (DCIS, 5.5% vs. IDC, 9.3%; *P* = 0.002). No significant difference was observed in the rate of locoregional recurrence between the 2 groups (*P* = 0.506). The prevalence of distant metastasis was significantly higher in the IDC group (*P* <  0.001) (Table 1).

**Table 1:** Clinicopathological characteristics of patients with breast cancer who were diagnosed with ductal carcinoma in situ and invasive ductal carcinoma

Characteristics	Ductal carcinoma in situ (*n* = 199)	Invasive ductal carcinoma (*n* = 914)	*P* value
Mean age (years, ±SD)	50.1 ± 9.4	49.3 ± 9.9	0.649
Mean body mass index (kg/m^2^, ± SD)	23.2 ± 3.0	23.4 ± 3.2	0.745
History of bilateral breast cancer (n, %)	14 (7.0)	30 (3.3)	0.014
Clinical tumor size on ultrasound (cm, ± SD)	2.01 ± 1.7	2.0 ± 1.2	0.213
Pathological tumor size (cm, ± SD)	2.2 ± 1.9	1.7 ± 1.1	0.109
Estrogen receptor, positive (n, %)	135 (67.8)	639 (69.9)	0.379
Progesterone receptor, positive (n, %)	120 (60.3)	568 (62.1)	0.136
c-erbB2 protein, positive (n, %)	73 (36.7)	169 (18.5)	0.226
Triple-negative breast cancer (n, %)	11 (5.5)	85 (9.3)	0.002
Adjuvant chemotherapy (n, %)	0	460 (50.3)	< 0.001
Adjuvant radiotherapy (n, %)	67 (33.7)	636 (69.6)	< 0.001
Adjuvant hormonal therapy (n, %)	125 (62.8)	687 (75.2)	0.061
Follow-up period (mo, ± SD)	90.1 ± 25.3	88.6 ± 19.1	0.241
Locoregional recurrence (n, %)	3 (1.5)	17 (1.9)	0.506
Distant metastasis (n, %)	1 (0.5)	24 (2.6)	< 0.001
Death (n, %)	1 (0.5)	9 (1.0)	0.192

**Fig. 2:**
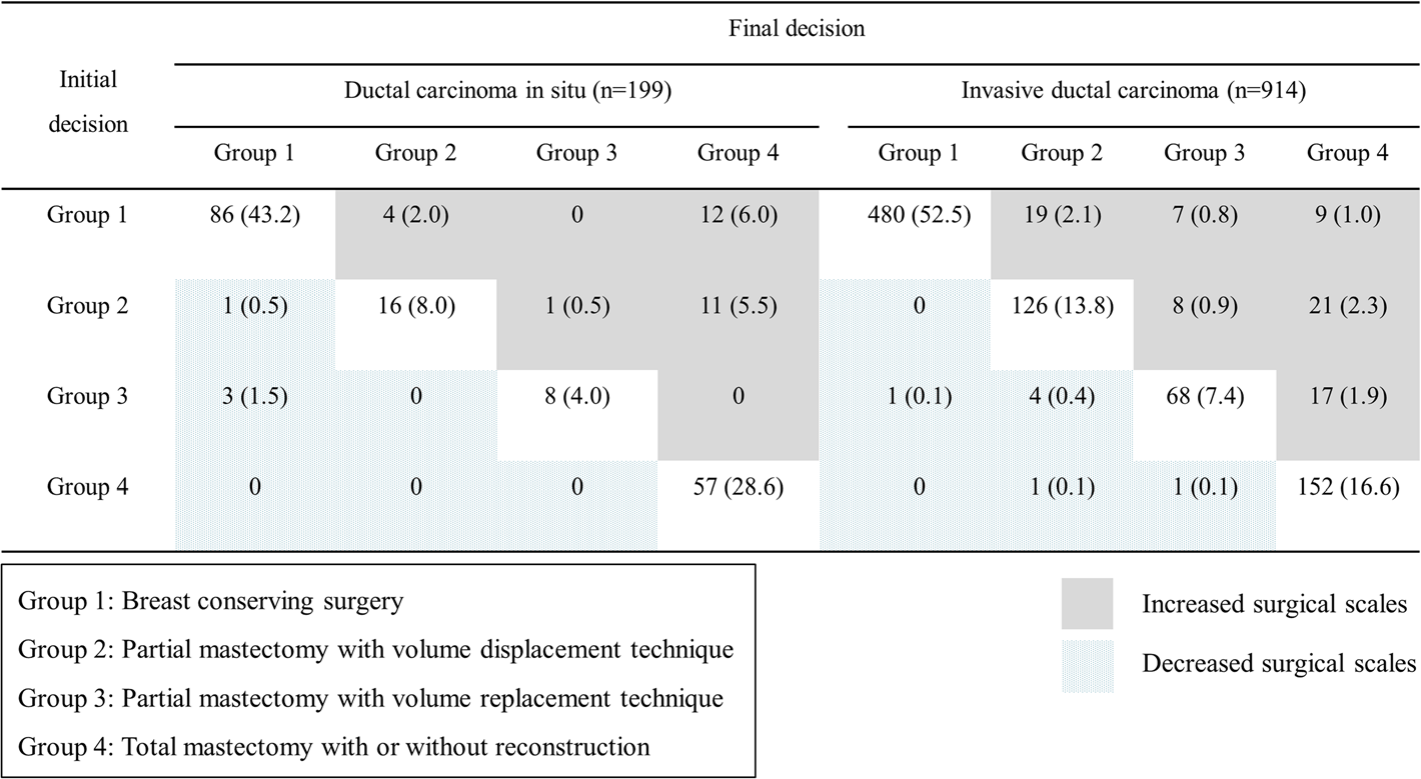
Group shifting of breast surgery based on breast magnetic resonance imaging findings of patients with ductal carcinoma in situ and invasive ductal carcinoma. The gray box represents the group with a higher surgical scale, and the dotted box represents the group with a lower surgical scale

The incidence of increased surgical scale was greater in the DCIS group (DCIS, 14.0% vs. IDC, 8.9%; *P* = 0.002). In addition, the incidence of decreased surgical scale was greater in the DCIS group (DCIS, 2.0% vs. IDC, 0.8%; *P* = 0.035) (Fig. 2). Therefore, significant differences were observed in the proportion of patients with changes in surgical plans between the DCIS and IDC groups (*P* <  0.001).

The increase in the surgical scale may be attributed to the following observations: increased extent of suspicious lesions on breast MRI compared with mammography and ultrasonography (DCIS, 9.0% vs. IDC, 7.1%), additional daughter nodules on breast MRI (DCIS, 3.5% vs. IDC, 1.4%), and multifocality or multicentricity on breast MRI (DCIS, 1.5% vs. IDC, 0.3%). However, the suspicious lesions detected on mammography or ultrasonography appeared benign on breast MRI. Hence, the surgical scale was decreased (DCIS, 2.0% vs. IDC, 0.8%). Six patients in the DCIS group and 4 patients in the IDC group requested to change the surgical plan regardless of imaging findings. However, in pathological evaluation, the incidence of positive margin in the initial frozen biopsy in Groups 1, 2, and 3 and nipple margin in Group 4 was not different between the 2 groups (*P* = 0.470 and 0.101) (Table 2).

**Table 2:** Changes in the surgical scale and reasons for the changes in the surgical plans of patients with ductal carcinoma in situ and invasive ductal carcinoma

	Ductal carcinoma in situ (*n* = 199)	Invasive ductal carcinoma (*n* = 914)	*P* value
Increased surgical scale (n, %)	28 (14.0)	81 (8.9)	0.002
Decreased surgical scale (n, %)	4 (2.0)	7 (0.8)	0.035
Total number of cases with surgical plan changes (n, %)	38 (19.1)	92 (10.1)	< 0.001
Changes in surgical plans based on MRI findings (n, %)	32 (16.1)	88 (9.6)	< 0.001
Reasons (n, %)			
Increased extent of suspicious lesions on breast MRI	18 (9.0)	65 (7.1)	–
Additional lesions on breast MRI	7 (3.5)	13 (1.4)	–
Multifocality or multicentricity on breast MRI	3 (1.5)	3 (0.3)	–
Suspicious lesions on mammography or ultrasonography but benign lesions on breast MRI	4 (2.0)	7 (0.8)	–
Changes in surgical plans due to patient’s desire	6 (3.0)	4 (0.4)	–
Positive margin status in the initial frozen biopsy (Groups 1–3)	13 (6.5)	59 (6.5)	0.470
Positive results in nipple frozen method (Group 4)	7 (3.5)	15 (1.6)	0.1301

*MRI* Magnetic resonance imaging

The pathological findings, which were matched to preoperative breast MRI findings, are shown in Table 3. Based on the final pathological reports, the number of cases showing a larger tumor size than that observed on mammography or ultrasonography was 14 (43.8%) in the DCIS group and 49 (55.7%) in the IDC group (*P* = 0.784). The pathological evaluation revealed true malignancy in DCIS (*n* = 9, 28.1%) and IDC (*n* = 32, 36.3%). However, among IDC cases showing true malignancy, the background of the DCIS component was observed in 27 cases (30.7%). In addition, benign pathological findings were found in DCIS (*n* = 5, 15.6%) and IDC (*n* = 17, 19.3%).

**Table 3:** Additional pathological results excluding the main lesion for cases with changes in the surgical plans based on MRI findings

Additional pathological results (n, %)	Ductal carcinoma in situ (*n* = 32)	Invasive ductal carcinoma (*n* = 88)	*P* value
Larger tumor size observed on breast MRI vs. breast mammography or ultrasonography	14 (43.8)	49 (55.7)	0.784
True malignancy	9 (28.1)	32 (36.3)	
Background of ductal carcinoma in situ (> 5 cm)	–	27 (30.7)	
Benign pathologic findings	5 (15.6)	17 (19.3)	
Multiple lymphovascular invasion	–	6 (6.8)	
Extensive intraductal component	–	9 (10.2)	
Multifocality	12 (37.5)	20 (22.7)	0.139
Separate nodules with invasive and non-invasive focus	–	3 (3.4)	
Microcalcification, both in tumor and benign ducts	6 (18.8)	18 (20.5)	0.950
Microcalcification, only in benign ducts	–	7 (8.0)	
Sclerosing adenosis or fibroadenoma	4 (12.5)	2 (2.3)	0.061

Factors could be duplicated

*MRI* Magnetic resonance imaging

In the IDC group, 6 (6.8%) cases involved multiple lymphovascular invasion and 9 (10.2%) demonstrated an extensive intraductal component. In the IDC group, the background of DCIS was found in 27 (30.7%) cases, and separate nodules with IDC and a noninvasive focus were found in 3 (3.4%) cases. The number of cases with microcalcifications, which were detected in both the tumor and benign ducts, was 6 (18.8%) in the DCIS group and 18 (20.5%) in the IDC group (*P* = 0.950). Several benign lesions were also detected in pathological findings.

Benign lesions including microcalcifications in benign ducts, sclerosing adenosis, and fibroadenoma were observed in 4 (12.5%) cases of DCIS and 9 (10.3%) cases of IDC (*P* = 0.061). In addition, these findings were matched to the suspicious lesions on preoperative breast MRI.

## Discussion

In comparison with mammography or ultrasonography, breast MRI is considered a superior imaging modality for assessing the extent of DCIS and detecting contralateral breast cancer during screening, which could improve the accuracy of therapeutic planning [15–17]. In particular, patients with pure DCIS may benefit more from breast MRI than from mammography, and this can be similarly useful when a DCIS component is combined with an IDC lesion [18].

However, based on hormonal or histopathological changes, the uptake of contrast in breast MRI could vary [19]. We have previously reported that breast MRI in triple-negative breast cancer is helpful for determining the surgical plan [14]. As a further study, we compared the changes in surgical plan between the IDC and DCIS groups, which were significantly more prevalent in the DCIS group.

Mammography is usually considered as a useful screening imaging modality for early breast cancer because clustered microcalcification is the most common mammographic finding among patients with DCIS [20, 21]. However, in non-mass-forming DCIS, which is common, the extent of the tumor is difficult to assess with only mammography or ultrasonography [22, 23]. Additional breast MRI may contribute to the accurate assessment of the extent of the DCIS focus or detection of occult breast cancer lesions in patients. As invasive breast cancer forms a mass or lump with typical malignant ultrasonographic features, including a hypoechoic mass with an irregular or spiculated margin, a lobulated shape with an indistinct margin, or a taller-than-wide shape [24], the extent of invasive breast cancer could be well identified by ultrasonography. However, when the DCIS component or extensive intraductal component is combined with invasive breast cancer, the extent of the breast cancer would be difficult to determine with mammography or ultrasonography.

The surgical plan for breast cancer can be established in more detail when more information is obtained from various images [25]. In current study, we found that the range of surgery, regardless increasing or decreasing, showed more changes in DCIS than IDC cases. Based on the pathophysiology of DCIS, the tumor starts from the ductal epithelium and tends to grow according to the ductal pattern. Therefore, DCIS usually appears as a clumped or linear enhancement or a non-mass enhancement on breast MRI without definite mass formation [20]. A recent study found that breast MRI was considerably more helpful in determining the surgical plan for the DCIS group. Additional breast MRI provided more information for surgical decision not only in cases with a higher surgical scale, but also in cases with a lower surgical scale. This finding suggests that compared with mammography or ultrasonography, breast MRI can better differentiate between benign and suspicious lesions in DCIS.

Based on several reports in the literature, the positive margin rate is significantly lower in cases in which preoperative MRI was conducted for breast cancer [1, 25]. However, in case the surgical margin is revealed as positive during the surgery, most surgeons would perform additional excision until a negative margin is confirmed. Therefore, in those cases, the oncological results would not be different in groups with MRI verses those without.

Occasionally, benign lesions appear similar to suspicious lesions on breast MRI. In a recent study, microcalcifications in benign ducts or sclerosing lesions including adenoma and fibroadenoma showed a similar pattern to that of malignant lesions on preoperative breast MRI. As a result, excision surgery was performed in these cases. However, there is no specific method to differentiate between a true malignant lesion and a benign lesion that could appear suspicious on breast MRI. Further studies are needed to improve the accuracy of differentiating these lesions before determining the surgical plan.

Although preoperative breast MRI can provide more information for determining the surgical range with high sensitivity, some investigators do not recommend breast MRI as a diagnostic imaging modality for breast cancer. Moreover, the detection rate can vary widely (40–100%) [26–31]. Therefore, breast MRI requires an experienced radiologist who can accurately interpret the images and determine the degree of suspicion of background breast parenchyma and contralateral breast parenchyma.

There are several limitations in current study. The number of DCIS cases was much lower than that of IDC cases. In addition, the surgical plans were decided not only reflect the information from images, but also based on the opinion of the patient through the discussion.

However, for breast cancer diagnosed as DCIS on needle biopsy and showing indistinct margins on mammography or ultrasonography, additional breast MRI is helpful for determining the surgical plan. In addition, it is beneficial in cases of invasive breast cancer with an intraductal component.

## Conclusion

Additional breast MRI could be more useful in determining the surgical plan for patients with DCIS than for those with IDC. In addition, this method would be useful for patients diagnosed with IDC with background DCIS components. However, our findings do not suggest that breast MRI should be performed for all patients with breast cancer. In further studies, investigators need to determine which patients can benefit most from breast MRI.

## Data Availability

The datasets generated and/or analyzed during the current study are not publicly available. However, they are available from the corresponding author on reasonable request.

## Abbreviations

DCIS: Ductal carcinoma in situ
IDC: Invasive ductal carcinoma
MRI: Magnetic resonance imaging
